# Ureteroarterial Fistula Secondary to External Iliac Artery Pseudoaneurysm in a Patient With Metastatic Colon Cancer

**DOI:** 10.7759/cureus.109025

**Published:** 2026-05-17

**Authors:** Stephen Patacsil, Brett McKeon

**Affiliations:** 1 Radiology, HCA Florida Aventura Hospital, Aventura, USA

**Keywords:** computed tomography angiography, endovascular stent graft, external iliac artery pseudoaneurysm, hematuria, ureteroarterial fistula

## Abstract

Ureteroarterial fistula is an uncommon yet potentially fatal source of hematuria, typically arising in patients with a history of pelvic surgery, radiation exposure, or malignancy.

An 80-year-old woman with a history of metastatic colon cancer status post chemoradiation and partial colectomy with Hartmann pouch formation, with bladder involvement, presented with abdominal pain and hematuria. During hospitalization, she developed hypotension with an acute drop in hemoglobin to 7 g/dL. Triphasic CT angiography (CTA) revealed active arterial contrast extravasation from the left external iliac artery into the distal ureter, consistent with a ureteroarterial fistula. Digital subtraction angiography (DSA) confirmed a left external iliac artery pseudoaneurysm, and the patient underwent successful endovascular management with a covered stent placement and exclusion of the left hypogastric artery. Hematuria resolved following intervention, with stabilization of hemodynamics and hemoglobin levels. The patient was maintained on continuous bladder irrigation. Although her long-term prognosis remained poor due to advanced malignancy, she was ultimately stabilized and discharged home.

This case emphasizes the need for early recognition of ureteroarterial fistula in high-risk patients and underscores the value of multiphasic imaging and endovascular management.

## Introduction

Ureteroarterial fistula is an uncommon yet potentially fatal source of hematuria caused by a fistulous connection between the ureter and a nearby arterial vessel. Fewer than 300 cases have been reported in the literature [1]. It most frequently occurs in individuals with a history of pelvic surgery, radiation exposure, malignancy, or repeated ureteral instrumentation [2]. Symptoms are often vague and may present as intermittent hematuria, which can delay diagnosis and increase the risk of serious complications, including morbidity and mortality. Diagnosis is particularly challenging because bleeding may be episodic, resulting in initially negative imaging or endoscopic evaluation. Imaging is essential for diagnosis, with CT angiography (CTA) and catheter-based angiography playing central roles in identification and management.

We report a case of ureteroarterial fistula in a patient with metastatic colorectal cancer who had undergone prior chemoradiation and surgery, emphasizing the importance of early detection and timely endovascular treatment in high-risk populations.

## Case presentation

An 80-year-old female with a history of metastatic colon carcinoma status post chemoradiation and partial colectomy with Hartmann pouch, with known metastatic involvement of the urinary bladder, presented with abdominal pain and hematuria. She was admitted for management of a urinary tract infection with intermittent hematuria, and during hospitalization she developed worsening gross hematuria, hypotension, and an acute drop in hemoglobin to 7 g/dL concerning for active hemorrhage.

**Figure 1:**
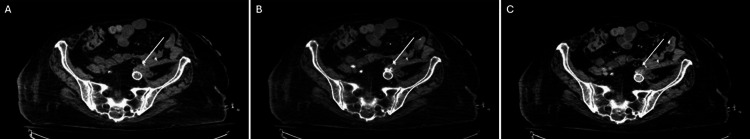
Triple-phase CTA Axial CT images of the abdomen demonstrating (A) noncontrast, (B) arterial phase, and (C) delayed phase imaging. The white arrow highlights a left external iliac artery pseudoaneurysm, best visualized on the arterial phase (B), with associated contrast extravasation into the adjacent distal ureter on delayed phase imaging (C). CTA: CT angiography

**Figure 2:**
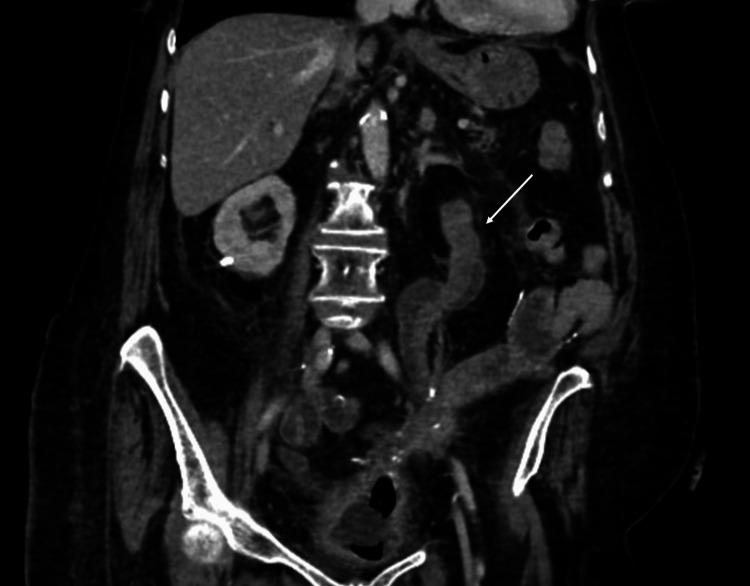
Coronal delayed-phase CT Coronal delayed-phase CT image of the abdomen demonstrating contrast extravasation into a dilated ureter (arrow), consistent with ureteroarterial fistula

Given suspicion for active hemorrhage, a triple-phase CTA, including noncontrast (Figure 1A), arterial phase (Figure 1B), and delayed phase imaging (Figure 1C), was performed. This revealed active contrast extravasation from the left external iliac artery into the ureter, raising strong concern for a ureteroarterial fistula (Figure 2).

**Figure 3:**
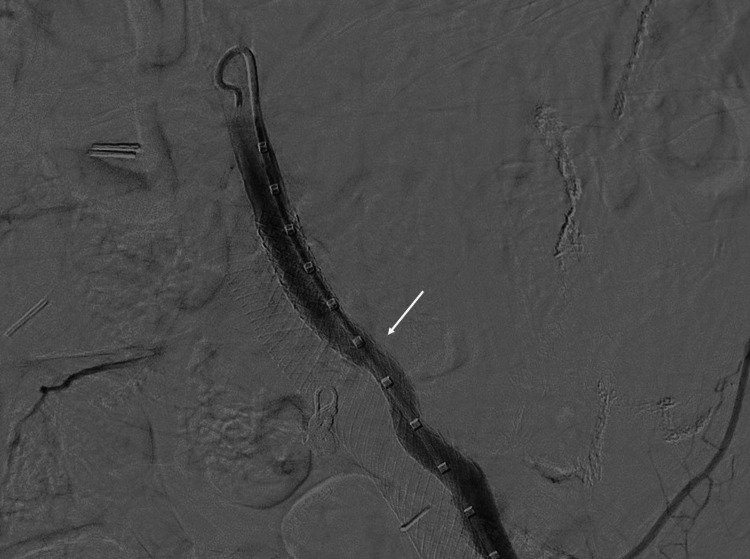
DSA DSA image demonstrating placement of a covered stent within the left external iliac artery, with exclusion of the previously identified pseudoaneurysm and no residual contrast extravasation DSA: Digital subtraction angiography

Given the imaging findings and ongoing clinical concern for hemorrhage, the patient subsequently underwent urgent digital subtraction angiography (DSA), which confirmed a pseudoaneurysm of the left external iliac artery. Endovascular treatment was performed with placement of a covered stent across the lesion, resulting in exclusion of the left hypogastric artery (Figure 3). 

Following the intervention, the patient's hematuria resolved, and her hemodynamic status and hemoglobin levels stabilized. She was maintained on continuous bladder irrigation. Although her long-term prognosis remained poor due to advanced malignancy, she was clinically stabilized and ultimately discharged home.

## Discussion

Ureteroarterial fistula represents a rare yet serious condition involving a fistulous communication between the ureter and an adjacent artery. Less than 300 cases have been reported in the literature, with significant morbidity and mortality largely attributable to the risk of massive bleeding [1]. It is most often encountered in patients with prior pelvic surgery, radiation exposure, malignancy, or chronic ureteral instrumentation [2].

The pathophysiology is thought to involve fibrosis and fixation of the ureter to adjacent vascular structures following surgery or radiation, with subsequent arterial pulsatility leading to erosion and fistula formation. In our patient, prior chemoradiation, pelvic surgery, and local tumor involvement of the bladder likely contributed to the development of an external iliac artery pseudoaneurysm with subsequent fistulization into the ureter.

Clinical presentation is often nonspecific and may include intermittent hematuria, which can delay diagnosis and increase the risk of life-threatening hemorrhage. This diagnostic difficulty highlights the need for a high level of clinical suspicion in patients at risk.

Imaging is central to establishing the diagnosis. CTA can reveal findings such as pseudoaneurysm formation, arterial contrast extravasation, or delayed contrast opacification of the ureter, though sensitivity may be reduced in the setting of intermittent bleeding. DSA remains the gold standard and also enables concurrent therapeutic intervention [3]. In this case, multiphasic CTA showed active extravasation from the external iliac artery into the distal ureter, which was subsequently confirmed as a pseudoaneurysm on angiography.

Endovascular management with covered stent placement has become the preferred treatment approach, offering a minimally invasive alternative to open surgical repair, which is associated with higher morbidity [4]. Successful exclusion of the pseudoaneurysm and fistula typically results in rapid resolution of hematuria, as demonstrated in our patient. However, open surgical repair remains an important option in cases of persistent or recurrent bleeding following attempted endovascular treatment [5].

## Conclusions

Ureteroarterial fistula should be suspected in patients presenting with hematuria and a history of pelvic surgery, radiation, or malignancy. Early recognition with multiphasic imaging and prompt endovascular management are essential to reduce morbidity and mortality. Given the rarity of ureteroarterial fistula, management should be individualized based on patient comorbidities, clinical stability, and available institutional expertise.
